# Lung Cancer Recurrence Risk Prediction through Integrated Deep Learning Evaluation

**DOI:** 10.3390/cancers14174150

**Published:** 2022-08-27

**Authors:** Peng Huang, Peter B. Illei, Wilbur Franklin, Pei-Hsun Wu, Patrick M. Forde, Saeed Ashrafinia, Chen Hu, Hamza Khan, Harshna V. Vadvala, Ie-Ming Shih, Richard J. Battafarano, Michael A. Jacobs, Xiangrong Kong, Justine Lewis, Rongkai Yan, Yun Chen, Franck Housseau, Arman Rahmim, Elliot K. Fishman, David S. Ettinger, Kenneth J. Pienta, Denis Wirtz, Malcolm V. Brock, Stephen Lam, Edward Gabrielson

**Affiliations:** 1 Department of Oncology, Johns Hopkins University, Baltimore, MD 21205, USA; 2 Department of Biostatistics, Johns Hopkins University, Baltimore, MD 21205, USA; 3 The Sidney Kimmel Comprehensive Cancer Center at Johns Hopkins, Baltimore, MD 21205, USA; 4 Department of Pathology, Johns Hopkins University, Baltimore, MD 21287, USA; 5 Department of Pathology, University of Colorado Anschutz Medical Campus, Aurora, CO 80045, USA; 6 Johns Hopkins Physical Sciences Oncology Center, Baltimore, MD 21218, USA; 7 Department of Chemical and Biomolecular Engineering, Johns Hopkins University, Baltimore, MD 21218, USA; 8 Institute for NanoBioTechnology, Johns Hopkins University, Baltimore, MD 21218, USA; 9 Bloomberg–Kimmel Institute for Cancer Immunotherapy, Johns Hopkins University, Baltimore, MD 21205, USA; 10 Department of Radiology, Johns Hopkins University, Baltimore, MD 21218, USA; 11 Department of Electrical Engineering, Johns Hopkins University, Baltimore, MD 21218, USA; 12 Department of Surgery, Johns Hopkins University, Baltimore, MD 21218, USA; 13 Department of Diagnostic and Interventional Imaging, The University of Texas Health Science Center at Houston, Houston, TX 77030, USA; 14 Department of Ophthalmology, Johns Hopkins University, Baltimore, MD 21218, USA; 15 Intensive Care Unit, Howard University College of Medicine, Washington, DC 20059, USA; 16 Department of Mechanical Engineering, Johns Hopkins University, Baltimore, MD 21218, USA; 17 BC Cancer Research Institute, University of British Columbia, Vancouver, BC V5Z 1L3, Canada

**Keywords:** postoperative-stage IA NSCLC, artificial intelligent, biomarker, computer-aided diagnosis, tumor grade

## Abstract

**Simple Summary:**

Few significant advances have been made over recent decades in predicting lung cancer progression risk after complete surgical removal of tumor in stage IA non-small-cell lung cancers (NSCLCs). Although several biomarkers have shown some predictive value, it is unclear whether these markers add value to traditional TNM staging. We developed an integrated deep learning evaluation (IDLE) score to combine patient’s preoperative lung CT image findings and postoperative pathologic assessment and found that this score can better predict cancer progression risk than TNM staging and tumor grade. Improved predictive value of the IDLE score was primarily due to the complementary use of tumor measurements in CT images from an entire lung as well as microscopic tissue characteristics. Our findings suggest that integrating measurements from different aspects of tumor morphology is more robust for increasing prediction accuracy than building on the measurements of similar aspects of tumor morphology.

**Abstract:**

*Background*: Prognostic risk factors for completely resected stage IA non-small-cell lung cancers (NSCLCs) have advanced minimally over recent decades. Although several biomarkers have been found to be associated with cancer recurrence, their added value to TNM staging and tumor grade are unclear. *Methods:* Features of preoperative low-dose CT image and histologic findings of hematoxylin- and eosin-stained tissue sections of resected lung tumor specimens were extracted from 182 stage IA NSCLC patients in the National Lung Screening Trial. These features were combined to predict the risk of tumor recurrence or progression through integrated deep learning evaluation (IDLE). Added values of IDLE to TNM staging and tumor grade in progression risk prediction and risk stratification were evaluated. *Results:* The 5-year AUC of IDLE was 0.817 ± 0.037 as compared to the AUC = 0.561 ± 0.042 and 0.573 ± 0.044 from the TNM stage and tumor grade, respectively. The IDLE score was significantly associated with cancer recurrence (*p* < 0.0001) even after adjusting for TNM staging and tumor grade. Synergy between chest CT image markers and histological markers was the driving force of the deep learning algorithm to produce a stronger prognostic predictor. *Conclusions:* Integrating markers from preoperative CT images and pathologist’s readings of resected lung specimens through deep learning can improve risk stratification of stage 1A NSCLC patients over TNM staging and tumor grade alone. Our study suggests that combining markers from nonoverlapping platforms can increase the cancer risk prediction accuracy.

## 1. Introduction

With the implementation of lung cancer screening using low-dose computed tomography worldwide, the proportion of stage IA lung cancers has increased to ≥50% compared to <10% in clinically diagnosed patients [1,2,3]. Stage IA non-small-cell lung cancer (NSCLC) patients are primarily treated with surgery alone, and patients with tumors that are completely resected with negative margins typically do not receive additional treatment. However, despite complete resection of the original tumor, cancer progression (recurrence, metastasis, or death) occurs in about 20% of patients with stage IA NSCLCs [4], and 70–90% of those who progress die from their lung cancers [5,6,7,8,9,10,11]. Successes in improving the outcomes of patients with IB or higher-stage resectable NSCLCs using neoadjuvant or adjuvant targeted therapy or immunotherapy alone or in combination with chemotherapy [12,13,14] lead to an increased interest in identifying the subset of stage IA NSCLC patients who may benefit from these therapies to improve the cure rate further. Currently, there are no validated prognostic markers to guide adjuvant therapy for patients with resected stage IA NSCLCs. 

Early identification of stage IA NSCLCs that have a high risk of progression has an important role for guiding physicians to proactively treat patients who may benefit from early cancer treatment to offset development of metastatic disease. While a number of promising blood or radiological biomarkers are found to be associated with NSCLC progression, none of them has shown significant added value over the well-established TNM staging and tumor grade criteria for stratification of lung cancer recurrence and mortality risk [15,16]. Since cancer progression is a complex process that involves multiple factors before and after surgery, we investigated integrated deep learning evaluation (IDLE) of tumor macro- and micromorphological characteristics for the potential to add value to TNM staging and tumor grade in identifying those stage IA NSCLCs that are at a high risk of cancer progression. 

Our report below is organized as follows. Section 2 describes how the study samples were selected, the method used to process images and extract features, the deep learning architecture, the statistical methods to evaluate predictors, and how we decoded the deep learning network’s black box to discover synergies between the lung low-dose computed tomography (LDCT) image markers and surgical tissue characteristics. The results are presented in Section 3. Study implications are discussed in Section 4. Finally, our conclusions are summarized in Section 5. Throughout the paper, we interchangeably used the terms “feature” and “variable” to refer to a risk factor under investigation, and we used “progression” to refer to the competing event of lung cancer recurrence, metastasis, and lung cancer-associated death.

## 2. Materials and Methods

### 2.1. Study Sample

**Figure 1:**
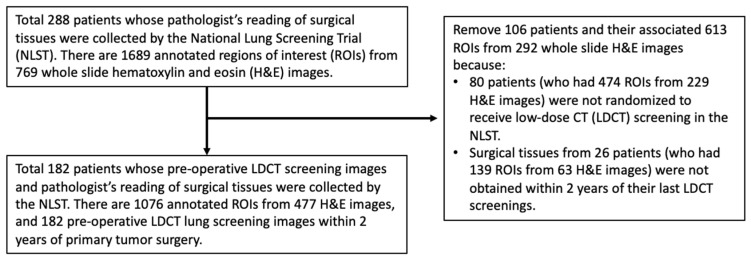
Study sample selection. Stage IA patients were selected from the National Lung Screening Trial who received primary tumor surgery within two years after their last LDCT screening date.

The National Lung Screening Trial (NLST) recruited 53,454 participants at a high risk of lung cancer between August 2002 and April 2004 from 10 clinical centers of the American College of Radiology Imaging Network (ACRIN) and 23 clinical centers of the Lung Screening Study group (LSS) [17]. The participants were randomized to receive either three annual LDCT screenings (T0, T1, T2) or annual X-ray screenings. From the surgical resection specimens of newly diagnosed tumors at the ACRIN centers, tissue blocks containing a formalin-fixed paraffin-embedded tumor, lymph nodes, and adjacent and distant lung tissue were forwarded to a central NLST pathology core laboratory. Multiple slides were then cut from each block, stained with hematoxylin and eosin (H&E), and imaged using an Aperio ScanScope. We received preoperative LDCT screening images, H&E section images, pathologist’s readings, and postoperative follow-up data from the NLST through several material transfer agreements. For this study, we selected NLST patients who met all the following criteria: (1) received primary lung tumor surgery; (2) had pathologist’s readings collected by the NLST; (3) had pathological stage IA NSCLC diagnosis according to the American Joint Committee on Cancer’s TNM staging system (8th edition) [18]; (4) had a largest pathological invasive tumor size no greater than 30 mm; and (5) had primary surgeries performed within 2 years after their last LDCT screening dates in the NLST study. A total of 182 patients met our selection criteria (Figure 1). From the pathology slides of these patients, 1076 pathologist-annotated regions of interest (ROIs) from 477 H&E images were identified, and 182 last preoperative LDCT lung screening images within 2 years of the primary tumor surgery were analyzed.

### 2.2. Tissue Image Feature Extraction 

Images of hematoxylin- and eosin (H&E)-stained sections from the National Lung Screening Trial (NLST) were stored on a server located at the University of Colorado. All H&E images were reviewed, and the regions of interest were annotated by the NLST reference pathologist (W.F.). These ROIs were selected from invasive tumor regions, pre-malignant regions (if present), and nontumor regions, and the predetermined features for each ROI were entered into an online database. All slides were reviewed by multiple pathologists to confirm accuracy of pathology readings, and the operation of the central NLST pathology core laboratory was summarized by Patz et al. [19]. We extracted tissue features from pathologist’s readings in all annotated ROIs associated with each individual patient. Five histologic subtype variables were created (Table S1) to classify tumors by diagnosis, which included adenocarcinoma in situ (AIS), invasive adenocarcinoma, carcinoid tumor, large-cell carcinoma, and squamous cell carcinoma. Table S1 lists all tissue features extracted from the annotated ROIs and surgical parameters.

### 2.3. Preoperative CT Image Feature Extraction

The CT scanner protocol parameters used in the NLST were 120 kVp, 40–80 mAs, detector collimation of 0.5–2.5 mm (for one data channel). All LDCT images were reconstructed through resampling and interpolation to have the same 0.5 mm × 0.5 mm × 0.5 mm voxel size. For the present study, we first segmented the whole lung to remove voxels from the bone and chest wall. All noncalcified lesions with diameters ≥ 4 mm were included in the analysis. Surgically resected tumors were linked to pathologic diagnosis, and lesions without pathological diagnosis were treated as indeterminant. To segment a tumor (or a lesion), we first defined its gross volume using a 3D cube that covered the entire tumor with at least 1 cm margin. Next, we applied a median filter to remove image noise before applying morphological operations to define tumor’s surface voxels. The median voxel intensity value from the adjacent normal lung tissue was subtracted from both tumor and peritumoral voxel values to normalize voxel intensities. We then added 300 to all voxel intensity values to make them non-negative, thus enabling radiomics energy features to quantify more effectively the lesion intensity distribution. 

Similar to our prior published work [20], we extracted LDCT image radiomics and other texture features from intra-tumor, peri-tumor, and extra-tumor volumes of interest (VOIs) (Table S2). A nested approach was used to extract voxel intensity spatial distribution. For this approach, we first used all voxels within the segmented lesion to define its weighted center as follows:

(1) $$C_{100}=(c_{x},\;c_{y},\;c_{z})=\left(\frac{\sum_{i,\;j,\;k}x_{ijk}d_{ijk}}{\sum_{i,\;j,\;k}d_{ijk}},\;\;\frac{\sum_{i,\;j,\;k}y_{ijk}d_{ijk}}{\sum_{i,\;j,\;k}d_{ijk}},\;\;\frac{\sum_{i,\;j,\;k}z_{ijk}d_{ijk}}{\sum_{i,\;j,\;k}d_{ijk}}\right)$$

where the summation is over all tumor voxels with coordinates $\left(x_{ijk},\;y_{ijk},\;z_{ijk}\right)$ and $d_{ijk}$ is the voxel intensity value at location $\left(x_{ijk},\;y_{ijk},\;z_{ijk}\right)$. The standard deviation of the tumor voxel spatial distribution was calculated by:

(2) $$LocSd_{100}=\sqrt{\frac{\sum_{ijk}d_{ijk}^{2}\left[{\left(x_{ijk}-c_{x}\right)}^{2}+{\left(y_{ijk}-c_{y}\right)}^{2}+{\left(z_{ijk}-c_{z}\right)}^{2}\right]}{\sum_{ijk}d_{ijk}^{2}}}\;$$

Since *LocSd*_100_ could be affected by tumor volume, we used *LocSd*_100_V = *LocSd*_100_/V to normalize *LocSd*_100_ where V is the tumor volume. We repeated the same formula using 50% and 20% of the voxels with the highest voxel intensity values to obtain the corresponding weighted centers (*C*_50_ and *C*_20_), *LocSd*_50_V, and *LocSd*_20_V. Distances between the centers were calculated by $d_{50}={∥C_{50}-C_{100}∥}_{2}$ and $d_{20}={∥C_{20}-C_{100}∥}_{2}$, where ${∥\cdot ∥}_{2}$ is the Euclidean distance metric.

The 3D radiomics features were extracted using formulas from Aerts et al. [21]. We used fixed bin size 100 (in Hounsfield units) to calculate energy, root-mean-square (RMS), entropy, and uniformity features. The second-order gray-level co-occurrence matrices (GLCMs) were calculated by setting directions *θ* = 0, 45, 90, and 135 in sagittal, transverse, and coronal planes, respectively. This resulted in 13 GLCMs from directions defined by the following vectors:

(1,0,0), (0,1,0), (1,1,0), (−1,1,0), (1,0,1), (1,1,1), (1,−1,1), (0,0,1), (0,1,1), (0,−1,1), (−1,0,1), (−1,1,1), (−1,−1,1).
(3)

For each of these 13 directions, the 22 GLCM texture features described in Table S2 were extracted. Their 3D texture feature values were then calculated using their average values over all 13 directions. For the second-order gray-level run-length matrix (GLRLM) feature extraction, we set the voxel distance parameter to *d* = 2, 3, 4, and 5, respectively, and used the 13 directions from (3) to calculate 52 (13 × 4) GLRLMs—one for each direction-by-distance combination. Eleven GLRLM texture features described in Table S2 were extracted from each GLRLM. Their 3D texture values were calculated using their average values over all 52 direction-by-distance combinations. 

**Figure 2:**
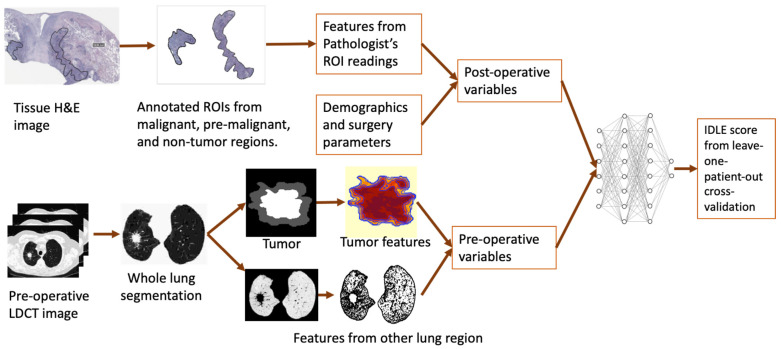
Study procedure. Postoperative variables include surgical tissue features extracted from pathologist’s readings, patient demographics, and surgical parameters. Preoperative variables include CT image features extracted from the tumor region, the peritumoral region, other area of the lung, and the time interval between preoperative LDCT lung scanning and surgery. All these variables were used as input variables in the leave-one-patient-out cross-validation IDLE score computation.

We used following features to compare intensity distributions between the voxels within the segmented lesions (or tumors) and the voxels from the peritumoral region:

(4) $$\mathrm{Mean}\;\mathrm{intensity}\;\mathrm{ratio}=\frac{\mathrm{mean}\;\mathrm{voxel}\;\mathrm{intensity}\;\mathrm{within}\;\mathrm{the}\;\mathrm{tumor}}{\mathrm{mean}\;\mathrm{voxel}\;\mathrm{intensity}\;\mathrm{within}\;\mathrm{the}\;\mathrm{peritumoral}\;\mathrm{region}}$$

(5) $$\mathrm{Quantile}\;\mathrm{ratio}\;R\left(q\right)=\frac{q\mathrm{th}\;\mathrm{voxel}\;\mathrm{intensity}\;\mathrm{quantile}\;\mathrm{within}\;\mathrm{the}\;\mathrm{tumor}\;}{q\mathrm{th}\;\mathrm{voxel}\;\mathrm{intensity}\;\mathrm{quantile}\;\mathrm{within}\;\mathrm{the}\;\mathrm{peritumoral}\;\mathrm{region}}$$

where *q* = 50 and 90, respectively. A total of 173 features from each LDCT image were extracted. The procedure to process images and extract features is illustrated in Figure 2. 

### 2.4. Prediction Algorithm Development 

We aimed to study the value of combining preoperative lung LDCT image texture features and histologic findings of resected tumors to predict the lung cancer progression risk using integrated deep learning evaluation (IDLE). The primary endpoint was progression-free survival defined as the time since the date of the initial primary tumor surgery to the date of lung cancer recurrence, metastasis, or lung cancer-related death, whichever came first. Patients who died from causes other than lung cancer or who were alive without progression were censored at the last contact date.

Although convolutional neural network (CNN) is a powerful tool for computer vision and image processing [22,23], a large sample size is generally required for a CNN to outperform handcrafted features [24]. For this reason, we chose to use a multilayer perceptron (MLP) neural network with handcrafted features because: (1) our study sample was not sufficiently large for CNN algorithm development; (2) it is difficult to decode the deep learning black box from automatically created CNN image features to identify the driving force that leads to the final predicted score. 

We used the following input variables for the IDLE (Figure 2): (1) patient demographics at the time of surgery, (2) surgery type (sublobar resection or lobectomy), (3) residual disease after surgery (R0 or R1), (4) lymphadenectomy received, (5) surgical tissue-associated features (listed in Supplementary Table S1), (6) preoperative LDCT lung image features (listed in Supplementary Table S2) in different anatomic lung locations, and (7) interval (in days) between the preoperative LDCT lung screening and surgery. We used MLP with two hidden layers and one last layer similar to our prior publication [25]. The first hidden layer activation functions were created from input variables, and the second hidden layer activation functions were created from the first hidden-layer variables with properly selected weights. The cross-entropy loss function with an L_2_ penalty parameter was used in feature selection and weight optimization in both hidden layers. The last layer used a random survival forest with input variables and weights coming from the second hidden layer. The final output of the network was from the random survival forest-predicted value normalized to between 0 and 1. The IDLE score was calculated as a predicted risk score through the leave-one-patient-out cross-validation method.

### 2.5. Statistical Methods

Summary statistics were used to compare demographics and clinical characteristics between the patients with and without progression. Two-sample *t*-test was used for continuous variables, and Fisher’s exact test was used for categorical variables. All tests were two-sided.

The prediction accuracy evaluation criteria included 5-year and 10-year area under the time-dependent ROC curve (AUC), time-dependent positive predictive value (PPV), time-dependent negative predictive value (NPV), and the hazard ratio (HR) of progression-free survival between high- and low-risk subgroups. The cutoff value of IDLE that maximizes the hazard ratio was selected to define its high-risk subgroup. The standard deviation of the AUC was computed using inverse probability of censoring weighted estimators in 500 bootstrap simulations. The time-dependent PPV was computed using Bayes’ rule:

(6) $$\mathrm{PPV}\left(t\right)=\frac{\left(\mathrm{time}-\mathrm{dependent}\;\mathrm{sensitivity}\;\mathrm{at}\;\mathrm{time}\;t\right)\times \left(1-S\left(t\right)\right)}{\mathrm{Positive}\;\mathrm{test}\;\mathrm{probability}}$$

where *S*(*t*) is the Kaplan–Meier estimate of progression-free survival probability at year *t*. The time-dependent NPV was calculated similarly. The added value of the IDLE scores to TNM staging and tumor grade was further evaluated through the multivariate Cox proportional hazards model adjusting for age at surgery, chemotherapy, and radiotherapy received.

### 2.6. Decoding the Deep Learning Black Box

To discover how features were used inside the deep learning black box, we took a slightly different approach from the leave-one-patient-out cross-validation method used by IDLE. We re-built IDLE networks using the same IDLE input variables but with all patients included (Figure S1A). We tracked how the input variables were processed within the network. We ranked the input features by the number of times they were selected across hidden layers, so the top ranked feature was defined as the most frequently selected variable. We then dichotomized these variables using the thresholds that maximize their hazard ratios in the univariate logrank test, with time to cancer progression as the endpoint. We repeated the same analysis using time to local recurrence and time to distant metastasis as the endpoint respectively. To find how deep learning enhanced features and how these features were integrated, we combined the input variables with all the hidden-layer features within the network and calculated their values using all 182 patients. Univariate tests were performed to examine how well each of these features was associated with cancer progression status using the *t*-test (for continuous features) and the chi-square test (for discrete features) to compare patients with and without lung cancer progression. Top 25 features with the smallest *p*-values from the *t*-test or the chi-square test were selected to construct a heatmap to visualize how these features worked together to boost prediction accuracy. We calculated *z*-scores for each feature using

(7) $$z=\frac{\left(\mathrm{feature}\;\mathrm{value}\right)-\left(\mathrm{mean}\;\mathrm{feature}\;\mathrm{value}\;\mathrm{across}\;\mathrm{all}\;\mathrm{patients}\right)}{\mathrm{standard}\;\mathrm{deviation}}$$

and plotted these *z*-scores on a heatmap.

To further evaluate synergy between preoperative LDCT image features and tissue H&E image features, we used exactly the same method for constructing an IDLE predictor to derive two additional deep learning predictors: the LDCT feature-derived predictor that did not use H&E features, and the H&E feature-derived predictor that did not use LDCT features. Their prediction accuracies were compared to IDLE through time-dependent ROC analyses. 

## 3. Results

### 3.1. Study Sample Characteristics

The demographics and clinical characteristics of the selected 182 patients are summarized in Table 1. During 12 years follow up, cancer progression was observed in 54 patients. Age at surgery, smoke pack-years, tumor location, surgery type, surgically removed lesion size, largest invasive tumor size, and TNM staging did not differ between the patients with and without cancer progression (all *p* > 0.10). However, in 39% (21/54) of the patients who progressed, cancers were diagnosed more than 6 months after their last LDCT screening date, compared to 21% (27/128) in the patients who did not progress (*p* = 0.0167). There was no statistical difference in the waiting time from the date of cancer diagnosis to the date of surgery between the patients with and without progression (*p* = 0.4042), but patients who progressed had a longer time interval between the last preoperative LDCT screening date and the surgery date (*p* = 0.0468). Surgery was the initial treatment for all patients, except for three patients who received chemotherapy or radiotherapy two to three months prior to surgery. Eighty-four percent (153/182) of patients received lobectomy, and 97% (177/182) of patients had no residual disease after surgery. Ninety percent (164/182) of patients also received lymphadenectomy with negative results. 

Each patient in our cohort had 2–3 digitally imaged H&E tumor sections, and a total of 477 H&E whole-slide images were available from all 182 patients. Within these H&E-stained slides, 1076 pathologist-annotated regions of interest (ROIs) were used to extract tissue features. Fifty-five patients had two or more histological subtypes reported in their H&E sections, including 53 patients with both adenocarcinoma and AIS; one patient with adenocarcinoma, squamous carcinoma, and AIS; and one patient with squamous carcinoma and AIS. The remaining 127 patients had only one histology subtype diagnosed, including 56 patients with adenocarcinoma, 15 patients with squamous carcinoma, 42 patients with large-cell carcinoma, and 14 patients with AIS. There was no statistical difference in the histological subtype distribution between the patients with and without progression (*p* = 0.1508), but patients who progressed did generally have higher tumor grades (*p* = 0.0163).

**Figure 3:**
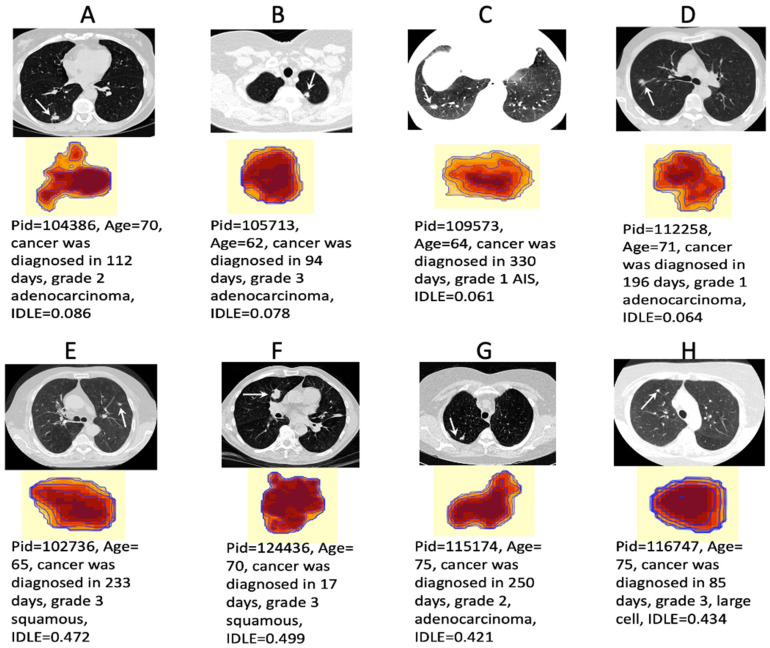
Examples of LDCT tumor texture maps and IDLE scores from four patients with nonaggressive tumors (**A**–**D**) who survived for over 10 years without cancer progression after the surgery and four patients with aggressive tumors (**E**–**H**) who died within 3 years after the surgery. Pid = patient ID number in the NLST.

The IDLE scores were developed through leave-one-patient-out cross-validation prediction. We found that patients who progressed had higher IDLE scores and a higher tumor grade than patients who did not progress. Figure 3 illustrates preoperative tumor LDCT image texture maps and the corresponding predicted IDLE scores from eight cancers. Patients shown in panels A, B, C, and D survived for over 10 years without lung cancer progression, whereas patients shown in panels E, F, G, H died from lung cancer within 3 years after surgery. All eight of these patients received primary tumor surgeries within one month of their cancer diagnoses. However, lung cancers were diagnosed 330, 233, and 250 days after the last low-dose CT screening dates for patients C, E, and G, respectively. We note that aggressive tumors (from patients E, F, G, and H) had substantially different LDCT image texture maps as compared to nonaggressive tumors (from patients A, B, C, and D) in voxel intensity and skewness patterns. 

### 3.2. Added Values of the IDLE Scores to TNM Staging and Tumor Grade

**Figure 4:**
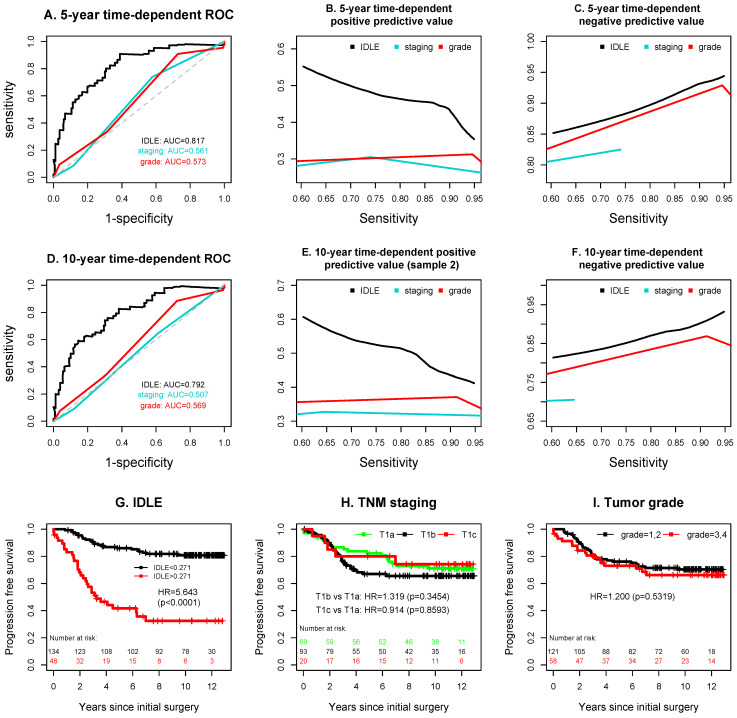
Prediction accuracy comparison of IDLE, TNM staging, and tumor grade. Negative predictive values for TNM staging were evaluable only in a part of sensitivity regions since higher sensitivities were not reached. The patients with undetermined (GX) tumor grade were excluded from the Kaplan–Meier curves in plot I.

Markedly increased prediction accuracy was observed with the application of IDLE scores. The 5-year time-dependent AUC of IDLE was 0.817 ± 0.037, which was significantly higher than the corresponding AUCs of 0.561 ± 0.042 and 0.573 ± 0.044 from TNM staging and tumor grade (Figure 4A). Similarly, the 10-year time-dependent AUC of IDLE was 0.792 ± 0.039 as compared to the AUCs of 0.507 ± 0.041 and 0.569 ± 0.045 from TNM staging and tumor grade (Figure 4D). The 5-year and 10-year time-dependent NPVs for TNM staging were evaluable only when sensitivities were less than 0.740 and 0.645, respectively, since higher sensitivities were not reached. After fixing the common 5-year and 10-year time-dependent sensitivities in the range of 60–95% for all predictors, IDLE resulted in uniformly higher positive predictive value and negative predictive value than TNM staging and tumor grade (Figure 4B,C,E,F). IDLE also better separated high risk subgroup from the low one (HR = 5.643, *p* < 0.0001) than TNM staging (T1b: HR = 1.319, *p* = 0.3454; T1c: HR = 0.914, *p* = 0.8593; T1a: reference) and tumor grade (HR = 1.200, *p* = 0.5319) (Figure 4G–I).

In the multivariate proportional hazards regression model that included IDLE, TNM staging, tumor grade, and the potential confounding variables (age at surgery, chemotherapy, and radiotherapy received either before or after the surgery) (Table 2), both TNM staging and tumor grade lost statistical association with cancer progression (*p* > 0.63) when IDLE was included in the model, while the association of IDLE with progression was highly significant (HR = 5.671, 95% CI = 3.1650–10.1605, *p* < 0.0001). Thus, IDLE has a stronger association with cancer progression than TNM staging and tumor grade in this multivariate analysis. 

### 3.3. Synergy inside the Deep Learning Network Black Box

The most frequently selected IDLE input variables by the deep learning hidden layers were (1) tumor grade > 1, (2) tumor *LocSdV*_100_ feature from the LDCT image, (3) tumor root-mean-square feature from the LDCT image, (4) age at surgery, (5) GLRLM skewness and kurtosis in the tumor’s LDCT image, (6) the largest invasive tumor dimension from the H&E image, (7) histology subtype other than AIS, and (8) days between the last LDCT screening date and the surgery date. The logrank test showed that these variables were able to significantly separate patient’s cancer progression risks with properly chosen cut points. Variables that were significantly associated with local recurrence or distant metastasis were only from the LDCT image features. Notably, tumor GLRLM skewness, solid or partially solid lesions in the right upper lobe, tumor region *LocSdV*_100_, and the tumor pixel mean and standard deviation values were all positively associated with local recurrence (*p*-values ranged from 0.0147 to 0.0425). The tumor pixel mean and root-mean-square values were also positively associated with distant cancer progression (*p* = 0.0007 and 0.0393). These observations imply that higher recurrence was observed among patients with solid or partially solid tumors as compared to nonsolid tumors, and their tumor voxel intensity distribution was skewed with one or more spatial clusters of high-intensity voxels.

When ranking the combined deep learning input and hidden-layer variables using *p*-values from the univariate *t*-test or the chi-square test to compare patients with and without cancer progression, all top 25 ranked features were from the hidden-layer variables that are composite functions of features in different platforms, including (1) preoperative LDCT image features, (2) surgical tissue features, (3) demographics, and (4) days between LDCT screening and surgery. Figure S1B shows that these hidden-layer variables, when working together, provided a better separation of patients with and without cancer progression than any single input variable, thus demonstrating strong synergy between the tumor’s global features (from preoperative LDCT images) and the localized histologic features (from H&E images).

To further study the synergy, we used exactly the same method of constructing IDLE to calculate LDCT feature-predicted scores (by excluding H&E features from the IDLE) and H&E feature-predicted scores (by excluding LDCT features from the IDLE). The 5- and 10-year time-dependent AUCs were 0.673 ± 0.041 and 0.632 ± 0.043 for the LDCT feature-predicted scores and 0.638 ± 0.050 and 0.648 ± 0.047 for the tissue H&E feature-predicted scores (Figure S2): they were all significantly lower than the AUCs from the IDLE. The time-dependent positive predictive values and negative predicted values (Figure S2) of these two individual scores were also uniformly lower than the corresponding integrated scores from the IDLE in Figure 4A–F.

## 4. Discussion

In this study, we showed that integrating diverse prognostic variables—global tumor features from preoperative LDCT images and localized histologic features from tissue H&E images—through a deep learning algorithm can identify aggressive stage IA NSCLCs better than TNM staging and tumor grade. Since IDLE provided uniformly higher ROC curve, positive predictive values, and negative predictive values than TNM staging and tumor grade, it has the potential to better identify patients with a high risk of cancer progression right after the primary surgery, and to select patients for early treatment. In this study, we demonstrated that the IDLE score added value to TNM staging and tumor grade and was significantly associated with cancer progression even after adjusting for the TNM stage and tumor grade (Table 2). These results imply that IDLE contains cancer progression risk information that is not available from the TNM stage and tumor grade. 

We identified the driving force of synergy between the global LDCT image features and the local tissue features inside the deep learning black box. These hidden-layer variables, when integrated together, were able to accurately differentiate the patients who had cancer progression from those who did not progress (Figure S1B). Interestingly, none of the individual input variables was among the top 25 ranked variables used by the deep learning network, implying that no individual input feature was sufficiently strong as a single marker. Even when combining multiple image features of LDCT or multiple morphological features of histopathology, the prediction accuracies of these separate approaches were substantially lower than the prediction accuracy of IDLE, which integrated both LDCT image features and histology image features (Figure S2 and Figure 4). 

Why does the integration of LDCT features with histology features provide a better predictive accuracy? While standardized tissue histopathological features provide insight into tumor-localized properties, histology of the tumor alone has limited ability to quantify how the tumor interacts with the global environment in the lung. Since LDCT image features quantify the tumor’s global morphology while H&E image features quantify the tumor’s local morphology, tumor characteristics from these two feature platforms are generally nonoverlapping. Our study suggests that integrating features from these nonoverlapping platforms produces a much stronger predictor than adding multiple features from a singular platform, which likely has overlapping information. Recently, a number of molecular biomarkers have been reported to have prognostic value in early lung cancer, and some of these markers also provide targets for treatment [26,27,28,29,30,31]. It is likely that integrating molecular biomarkers with IDLE may further improve prediction accuracy. To facilitate such an investigation, we make our IDLE data publicly available at GitHub with the participants’ NLST identification numbers included. 

Interestingly, the number of days between the last LDCT image scanning date and the surgery date was negatively associated with progression-free survival, and this variable was one of the top input features used by the deep learning network. A longer interval between the LDCT screening date and the surgery date was primarily due to the delay in cancer diagnosis. Among the 54 patients who progressed, 21 patients had delayed cancer diagnosis (Table 1), and even though they were still in stage IA at the time of surgery, they had a higher tumor grade than the other patients. Timely diagnosis of aggressive tumors is evidently important to improve the lung cancer screening efficacy. Our recent work in DeepLR [25] (available at www.caced.jhu.edu) provides estimates of lung cancer incidence risk, tumor aggressiveness, and suggests screening intervals that could help earlier diagnosis of aggressive tumors. 

TNM staging and tumor grade have long been recognized to be robust prognostic markers of cancer progression for postoperative NSCLC patients. Attempts to improve this clinical gold standard through computer-aided prediction models are sparse. D’Antonoli et al. showed that using a combination of tumoral and peritumoral radiomic features in preoperative CT with TNM staging outperformed TNM staging alone in patients with stage IA–IIB [32]. Wang et al. showed that the top nuclear morphometric features from H&E tissue microarray images were associated with cancer recurrence in patients with resected early-stage NSCLCs [33]. Other authors have used either CT features or histopathological features to identify patients with early-stage NSCLC who are at risk of local recurrence or distant metastasis [16,34,35]. Our study represents the first attempt that combines both LDCT and histopathology features to improve the prediction of cancer recurrence or progression. 

A limitation that we recognize in our study is that the sample size was relatively small, and the results were only cross-validated. Further prospective validation in a larger sample is needed. Recognizing this limitation and the need for further refinement of the IDLE score, our study does support our original hypothesis that nonoverlapping tumor morphological features from different platforms can improve lung cancer progression risk stratification over TNM staging and tumor grade.

## 5. Conclusions

Low-dose computed tomography (LDCT) screening for high-risk individuals increases the detection of stage IA NSCLCs [2,3]. The current standard of care is surgery alone. However, stage I NSCLCs treated in this manner have diverse survival outcomes [36,37,38,39,40]. Identifying the subset of patients with potentially curable lung cancer who are at a high risk of tumor recurrence or progression who would benefit from adjuvant therapy is critical for improving the outcome of these patients. Our study supports the use of diverse nonoverlapping tumor measures in lung cancer risk evaluation. The findings of our study have potential to not only help treating oncologists improve clinical management of early-stage NSCLCs, but also to help investigators to better define patient selection criteria and determine what measurements to collect in future clinical trial designs. 

## Figures and Tables

**Table 1:** Summary of the study sample. Cancer progression is defined as a competing event from any of the following events during the 12-year follow-up after the primary tumor surgery: lung cancer recurrence, metastasis, or lung cancer-related death.

		No Progression N = 128	Progression N = 54	*p* ^1^
Cancers diagnosed 6 months after the last LDCT screening date		27	21	0.0167
Lung cancer-related death		0	45	
Age at surgery		64.7 ± 4.9	65.9 ± 4.8	0.1067
Female, N (%)		58 (45%)	24 (44%)	1.0
Smoke pack-years		66 ± 29	72 ± 41	0.2889
Days from the last LDCT screening to the date of lung surgery		177 ± 210	267 ± 299	0.0468
Surgery type	Sublobar resection Lobectomy	21 107	8 46	1.0
Lymphadenectomy	N (%)	115 (90%)	49 (91%)	1.0
Residual disease after surgery	R0	124	53	1.0
	R1	4	1	
Surgically removed lesion size (mm)		19.7 ± 13.9	20.6 ± 12.6	0.6713
Largest invasive tumor size (mm)		11.4 ± 6.8	12.9 ± 6.4	0.1604
Pathological cancer stage (TNM, 8th edition)	IA1 (T1a)	50	19	
	IA1 (T1b)	63	30	1.0
	IA1 (T1c)	15	5	
Highest tumor grade from all the ROIs	1 = well-differentiated	34	4	0.0163
	2 = moderately differentiated	53	30	
	3 = poorly differentiated	35	14	
	4 = undifferentiated	5	4	
	Undetermined (GX)	1	2	

^1^ Two-sample *t*-test was used for continuous variables, and Fisher’s exact test was used for categorical variables. All tests were two-sided.

**Table 2:** Multivariate analysis to study the added value of IDLE.

	HR	95% CI	*p*
IDLE high	5.6708	(3.1650, 10.1605)	<0.0001
T1b ^1^	0.8665	(0.4667, 1.6087)	0.6499
T1c ^1^	0.7708	(0.2620, 2.2680)	0.6364
High grade	0.9818	(0.5440, 1.7720)	0.9513
Age at surgery	1.0318	(0.9738, 1.0934)	0.2888
Chemotherapy	0.6700	(0.2806, 1.5996)	0.3671
Radiotherapy	1.3959	(0.4064, 4.7945)	0.5963

^1^ The reference is the T1a subgroup.

## Data Availability

Source data, extended data, and code to analyze the extended data are available at https://github.com/ph202203/stage-IA-NSCLC.

## Supplementary Materials

The following supporting information can be downloaded at: https://www.mdpi.com/article/10.3390/cancers14174150/s1, Figure S1: Decoding synergy among input features within the deep learning black-box (A) and heatmap from top features inside the IDLE black-box (B). Figure S2: Prediction accuracies from preoperative low-dose CT (LDCT) image feature-derived scores and surgical tissue H&E image feature-derived scores. Table S1: Surgical tissue histopathological features and clinical variables extracted from the National Lung Screening Trial (NLST) database. Table S2: Radiomics and other texture features extracted from preoperative low-dose CT screening images.
