# Supplementary material for: Lung Cancer Recurrence Risk Prediction through Integrated Deep Learning Evaluation
Source: Cancers (Basel). 2022 Aug 27;14(17):4150. doi: 10.3390/cancers14174150 (PMC9454871; doi:10.3390/cancers14174150)

Supplementary tables and figures

For "Lung cancer recurrence risk prediction through integrated deep learning evaluation" by

Peng Huang [1,2,3,*], Peter B. Illei [3,4], Wilbur Franklin [5], Pei-Hsun Wu [6,7,8], Patrick M. Forde [1,3,9], Saeed Ashrafinia [10,11], Chen Hu [1,2,3], Hamza Khan [12], Harshna V. Vadvala [10], Ie-Ming Shih [3,4], Richard J. Battafarano [3,12], Michael A. Jacobs [3,10,13], Xiangrong Kong [2,14], Justine Lewis [15], Rongkai Yan [10], Yun Chen [16], Franck Housseau [1,3,9], Arman Rahmim [10,11,17], Elliot K. Fishman [1,3,10,12], David S. Ettinger [1,3], Kenneth J. Pienta [1,3], Denis Wirtz [1,3,6,7,8], Malcolm V. Brock [3,12], Stephen Lam [17,†] and Edward Gabrielson [3,4,†]

Table S1. Surgical tissue histopathological features and clinical variables extracted from National Lung Screening Trial (NLST) database.

Feature category	Features
Clinical measure	Lesion size (mm) in resected lung specimen,<br>Surgery type= (Sublobar resection; lobectomy)<br>Residual disease after the surgery (R0, R1)<br>Lymphadenectomy
Tumor regions	Regional highest tumor grade<br>Regional lymphoid infiltration<br>Regional vessel invasion<br>Highest regional tumor cell percent<br>Highest regional inflammatory cell percent<br>AIS = ("AIS Mixed Non-mucinous/Mucinous"; "AIS Mucinous"; "AIS Non-mucinous"; "AIS NOS or Indeterminate")<br>Adenocarcinoma = ("Solid Adenocarcinoma with mucin production"; "Solid Adenocarcinoma with mucin production; Clear Cell and Acinar"; "Acinar Adenocarcinoma"; "Adenocarcinoma Micropapillary"; "Adenocarcinoma, Mixed Subtype"; "Adenocarcinoma, NOS"; "Adenosquamous Carcinoma"; "Fetal Adenocarcinoma"; "Mucinous colloid carcinoma"; "Mucinous cystadenocarcinoma"; "Papillary Adenocarcinoma")<br>Carcinoid = ("Carcinoid Tumor; Atypical"; "Carcinoid Tumor, Typical"; "Carcinoid tumorlet")<br>Large Cell = ("Carcinoma NOS"; "Giant Cell Carcinoma"; "Large Cell Carcinoma"; "Large Cell Carcinoma with Rhabdoid Phenotype"; "Spindle Cell carcinoma")<br>Squamous = ("Squamous Cell Carcinoma"; "Squamous Cell Carcinoma, Basaloid"; "Squamous Cell Carcinoma, Clear Cell"; "Squamous Cell Carcinoma, Papillary")
Non-tumor regions	Chronic bronchitis<br>Emphysema<br>Fibrosis<br>Granuloma<br>Hemorrhage<br>Infarction<br>Necrosis<br>Pneumonia
Pre-malignant regions	"Squamous Dysplasia, MODERATE";<br>"Squamous dysplasia, SEVERE";<br>"Squamous Cell Carcinoma In Situ";<br>"None"

Table S2. Radiomics and other texture features extracted from pre-operative low-dose CT screening images. Radiomics features were calculated using formulas from Aerts et al [21].

Feature category	Features
First order statistics from within and peri-nodule regions	mean, median, variance, quantiles, skewness, kurtosis, energy, entropy, root mean square, uniformity.
Shape and volume from within and peri-nodule regions	compactness, maximum 3D diameter, sphericity, surface area, volume, surface to volume ratio
Gray level co-occurrence matrix (GLCM) texture features from within and peri-nodule regions	Autocorrelation, cluster prominence, cluster shade, cluster tendency, contrast, correlation, difference entropy, dissimilarity, energy, entropy, homogeneity 1 and 2, information measure of correlation 1 and 2, inverse difference moment normalized, inverse difference normalized, inverse variance, maximum probability, sum average, sum entropy, sum variance, variance.
Gray level run-length matrix (GLRLM) texture features from within and peri-nodule regions	Short run emphasis, long run emphasis, gray level non-uniformity, run length non-uniformity, run percentage, low gray level run emphasis, high gray run emphasis, short run low gray level emphasis, short run high gray level emphasis, long run low gray level emphasis, long run high gray level emphasis.
Contrast from within and peri-nodule regions	Ratios of mean intensities and quantiles between voxels inside the tumor and voxels in peri-tumor area
Voxel intensity spatial distribution from within the nodule	$LocSd_{100}$, $LocSdV_{100}$, $LocSd_{50}$, $LocSdV_{50}$, $LocSd_{20}$, $LocSdV_{20}$, d_{50}, d_{20}
Tumor and other lesion location	RUL, RML, RLL, LUL, Lingular, LLL, carina, left hilum, right hilum, left main stem bronchus, right main stem bronchus, mediastinum, others
Emphysema	Lung median density and a binary indicator from the NLST data
Cardiovascular abnormality	A binary indicator from the NLST data

Figure S1. Decoding synergy among input features within the deep learning black-box (A) and heatmap from top features inside the IDLE black-box (B).

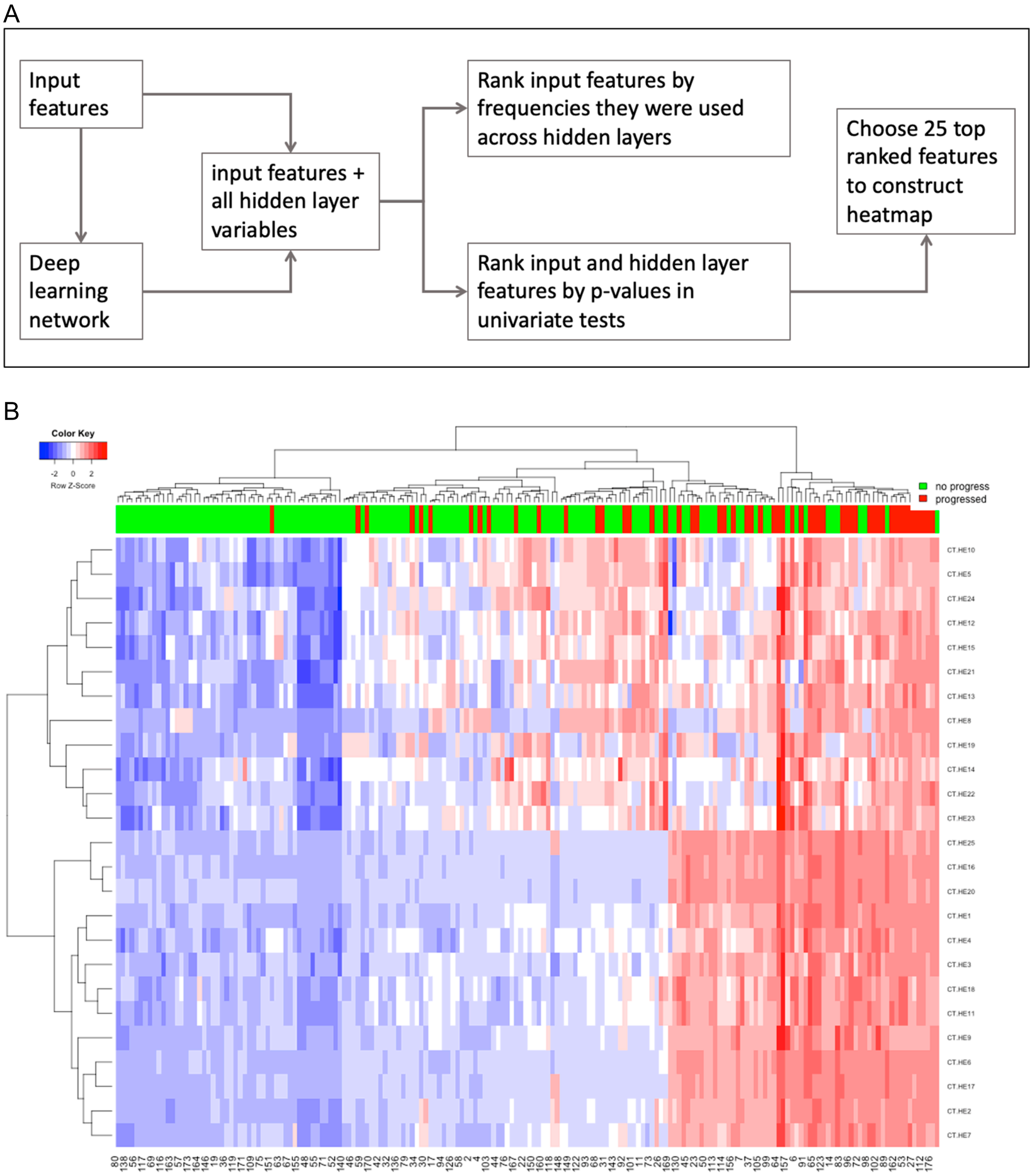

Figure S2. Prediction accuracies from pre-operative low-dose CT (LDCT) image feature-predicted scores (by excluding H&E features from the IDLE) and H&E feature-predicted scores (by excluding LDCT features from the IDLE). The method used to calculate these scores is the same as the method used to calculate IDLE scores, i.e., using leave-one-patient-out cross-validation.

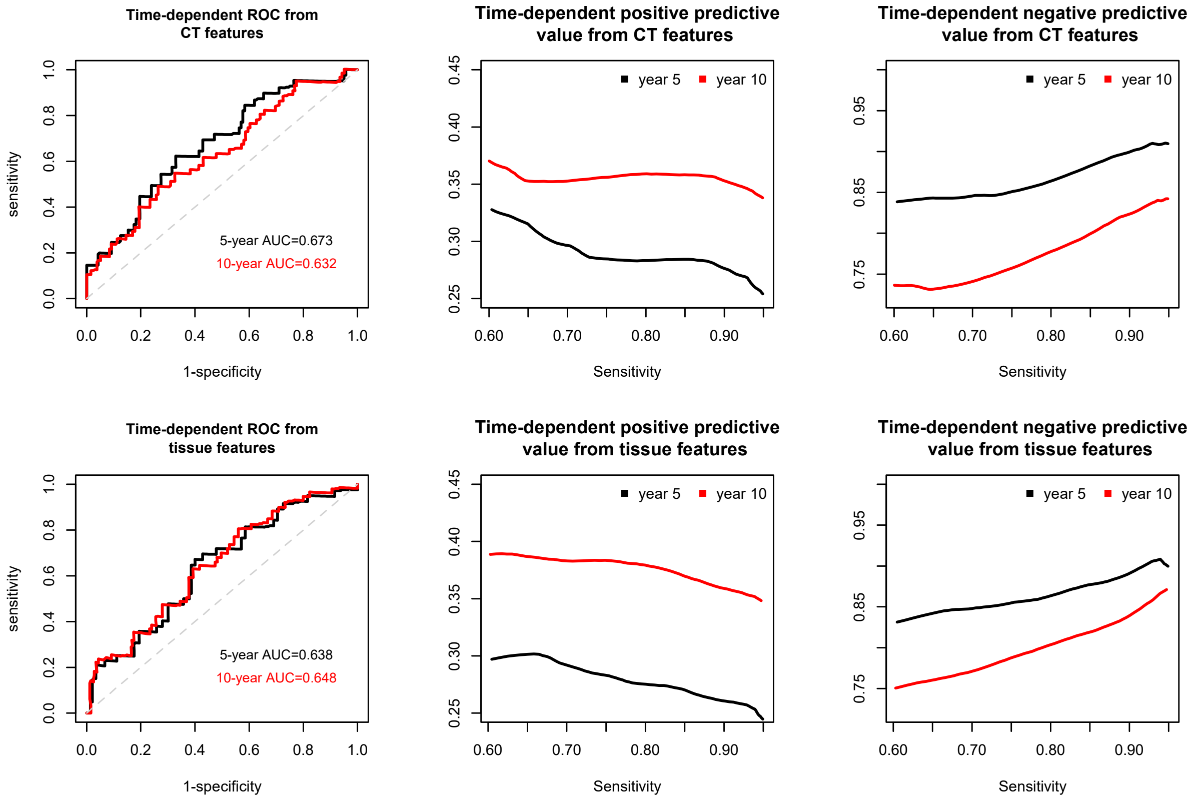

Supplement: Supplementary file 1 [file cancers-14-04150-s001.zip › cancers-1864495-supplementary.pdf]
